# Association of CLDN6 and CLDN10 With Immune Microenvironment in Ovarian Cancer: A Study of the Claudin Family

**DOI:** 10.3389/fgene.2021.595436

**Published:** 2021-06-23

**Authors:** Peipei Gao, Ting Peng, Canhui Cao, Shitong Lin, Ping Wu, Xiaoyuan Huang, Juncheng Wei, Ling Xi, Qin Yang, Peng Wu

**Affiliations:** ^1^Cancer Biology Research Center (Key Laboratory of the Ministry of Education), Department of Obstetrics and Gynecology, Tongji Hospital, Tongji Medical College, Huazhong University of Science and Technology, Wuhan, China; ^2^Department of Obstetrics and Gynecology, Tongji Hospital, Tongji Medical College, Huazhong University of Science and Technology, Wuhan, China; ^3^Institute of Pathology, Tongji Hospital, Tongji Medical College, Huazhong University of Science and Technology, Wuhan, China

**Keywords:** ovarian cancer, CLDN6, CLDN10, prognosis, immune microenvironment

## Abstract

**Background:**

The claudin family is a group of transmembrane proteins related to tight junctions. While their involvement in cancer has been studied extensively, their relationship with the tumor immune microenvironment remains poorly understood. In this research, we focused on genes related to the prognosis of ovarian cancer and explored their relationship with the tumor immune microenvironment.

**Methods:**

The cBioPortal for Cancer Genomics database was used to obtain the genetic variation pattern of the claudin family in ovarian cancer. The ONCOMINE and Gene Expression Profiling Interactive Analysis (GEPIA) databases were used to explore the mRNA expression of claudins in cancers. The prognostic potential of these genes was examined via the Kaplan-Meier plotter. The enrichment of immunological signatures was determined by gene set enrichment analysis (GSEA). The correlations between claudins and the tumor immune microenvironment in ovarian cancer were investigated via the Tumor Immune Estimation Resource (TIMER).

**Results:**

Claudin genes were altered in 363 (62%) of queried patients/samples. Abnormal expression levels of claudins were observed in various cancers. Among them, CLDN3, CLDN4, CLDN6, CLDN10, CLDN15, and CLDN16 were significantly correlated with overall survival in patients with ovarian cancer. GSEA revealed that CLDN6 and CLDN10 were significantly enriched in immunological signatures of B cell, CD4 T cell, and CD8 T cell. Furthermore, CLDN6 and CLDN10 were negatively correlated and positively correlated, respectively, with immune cell infiltration in ovarian cancer. The expression levels of CLDN6 and CLDN10 were also negatively correlated and positively correlated, respectively, with various gene markers of immune cells in ovarian cancer. Thus, CLDN6 and CLDN10 may participate in immune cell infiltration in ovarian cancer, and these mechanisms may be the reason for poor prognosis.

**Conclusion:**

Our study showed that CLDN6 and CLDN10 were prognostic biomarkers correlated with the immune microenvironment in ovarian cancer. These results reveal new roles for CLDN6 and CLDN10 as potential therapeutic targets in the treatment of ovarian cancer.

## Introduction

Ovarian cancer is the most lethal gynecological cancer among women (Siegel et al., 2020). Although surgical techniques and combined chemotherapy applications have progressed since the 1970s, the 5 year survival rate of advanced ovarian cancer is only 40–45% (Henderson et al., 2018). Therefore, improved treatment of ovarian cancer remains an urgent issue. Immunotherapy is an emerging treatment for several solid tumors, which shows improved outcomes in patients. With the application of various immune-based interventions in ovarian cancer, immunotherapy has been proven useful in advanced disease (Bogani et al., 2020).

The claudin (CLDN) family consists of more than 20 transmembrane proteins, which are major components of tight junctions. They serve as a physical barrier to prevent molecules from passing freely through the paracellular space between epithelial or endothelial cell sheets and also play critical roles in maintaining cell polarity and signal transductions (Weinstein et al., 1976; Wodarz, 2000; Tsukita et al., 2001; Kirschner et al., 2013). Previous research has recognized various claudin gene expression patterns and identified several genes dysregulated in cancers (Hewitt et al., 2006). These genes play roles in the tumorigenesis of solid tumors (Swisshelm et al., 2005; Hagen, 2019) and represent promising targets for cancer detection, prognosis, and therapy (Morin, 2005). However, the relationship between claudins and the tumor immune microenvironment has not yet been elucidated. This study comprehensively analyzed claudin expression in ovarian cancer and further explored the relationship between claudins and the immune microenvironment.

## Materials and Methods

### cBioPortal

The cBioPortal database^1^ (Cerami et al., 2012; Gao et al., 2013) is an open platform for cancer genomics analysis. In total, 585 samples of ovarian serous cystadenocarcinoma (The Cancer Genome Atlas (TCGA), Pan-Cancer Atlas) were used for genetic variation analyses through the cBioPortal.

### ONCOMINE Database Analysis

Claudin expression levels in various cancers were analyzed via the ONCOMINE database^2^ (Rhodes et al., 2007), which includes more than 35 types of cancer and normal samples.

### Gene Expression Profiling Interactive Analysis (GEPIA)

GEPIA v2^3^ (Tang et al., 2017) is used to analyze the RNA sequencing expression data of 9736 tumors and 8587 normal samples from the TCGA and GTEx projects using a standard processing pipeline. The expression profile of the claudins in ovarian cancer was explored via GEPIA v2. The *p*-value cutoff was 0.05 and | log_2_FC| cutoff was 1.5.

### Kaplan-Meier Plotter Database Analysis

The Kaplan-Meier plotter^4^ (Gyorffy et al., 2012) assesses the effects of 54,000 genes on survival in 21 cancer types. The largest datasets include breast (*n* = 6234), ovarian (*n* = 2190), lung (*n* = 3452), and gastric (*n* = 1440) cancer. The system includes gene chip and RNA-seq data-sources from the Gene Expression Omnibus (GEO), European Genome-Phenome Archive (EGA), and TCGA databases. The prognostic significance of claudins in ovarian cancer was analyzed via the online database.

### Tumor Immune Estimation Resource (TIMER)

TIMER^5^ (Li et al., 2017) allows comprehensive analysis of tumor-infiltrating immune cells. The correlation between claudin expression and immune cell infiltration was analyzed using this database. TIMER v2, an updated and enhanced version of TIMER, was used to analyze immune infiltration across diverse cancer types.

### Statistical Analyses

The expression levels of claudins are presented as mean ± standard deviation (SD). Kaplan-Meier survival curves were established based on the log-rank test. The hazard ratio (HR) was determined using the Cox model. Spearman correlation was used for correlation analysis. A *p*-value of < 0.05 was considered to be significant.

## Results

### Gene Variation of Claudins in Ovarian Cancer

**FIGURE 1:**
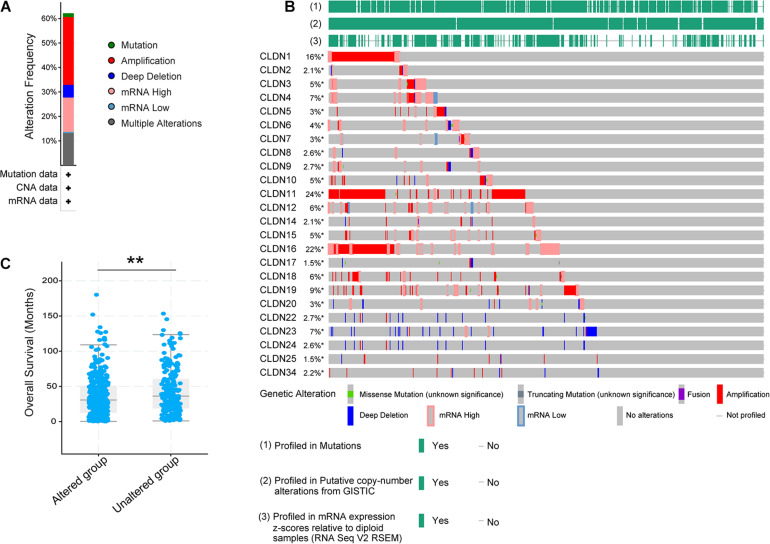
The genetic variation of the claudin family in ovarian cancer through the cBioPortal. **(A)** The alteration frequency of the claudin family in serous ovarian cancer. **(B)** The oncoprint of the claudin family in serous ovarian cancer. **(C)** The overall survival difference of serous ovarian cancer between the altered and unaltered group (**p < 0.01).

Twenty-four reviewed proteins of the claudin family were obtained from the UniProt Knowledgebase (UniProtKB)^6^ (Table 1) [an additional file shows this in more detail (see Table 1)]. Firstly, we investigated the genetic variation of the claudin family in ovarian cancer using the cBioPortal for Cancer Genomics. Twenty-four genes were queried in 585 samples of ovarian serous cystadenocarcinoma (TCGA, Pan-Cancer Atlas). Figure 1A shows the alteration frequency of genetic variation in serous ovarian cancer. As shown in Figure 1B, the queried genes were altered in 363 (62%) queried patients/samples. The top three gene variations were *CLDN11* (24%), *CLDN16* (22%), and *CLDN1* (16%). Differences in overall survival (OS) between the altered and unaltered groups were compared using the Kruskal-Wallis test. We found that OS was reduced in the altered group compared to the unaltered group (*p* = 7.981e-3) (Figure 1C). Previous studies have shown that the claudin family is dysregulated in a variety of tumors and is involved in diagnosis, tumorigenesis, and prognosis (Zhang et al., 2013; Barros-Filho et al., 2015; Zhou et al., 2018). Thus, the claudin family is worthy of further research in ovarian cancer.

**TABLE 1:** Twenty-four reviewed proteins of claudin family from the UniProtKB.

**Entry**	**Status**	**Gene names**	**Protein names**	**Organism**
O95832	Reviewed	CLDN1	Claudin-1 (Senescence-associated epithelial membrane protein)	Homo sapiens
P78369	Reviewed	CLDN10	Claudin-10 (Oligodendrocyte-specific protein-like) (OSP-like)	Homo sapiens
O75508	Reviewed	CLDN11	Claudin-11 (Oligodendrocyte-specific protein)	Homo sapiens
P56749	Reviewed	CLDN12	Claudin-12	Homo sapiens
O95500	Reviewed	CLDN14	Claudin-14	Homo sapiens
P56746	Reviewed	CLDN15	Claudin-15	Homo sapiens
Q9Y5I7	Reviewed	CLDN16	Claudin-16 (Paracellin-1) (PCLN-1)	Homo sapiens
P56750	Reviewed	CLDN17	Claudin-17	Homo sapiens
P56856	Reviewed	CLDN18	Claudin-18	Homo sapiens
Q8N6F1	Reviewed	CLDN19	Claudin-19	Homo sapiens
P57739	Reviewed	CLDN2	Claudin-2 (SP82)	Homo sapiens
P56880	Reviewed	CLDN20	Claudin-20	Homo sapiens
Q8N7P3	Reviewed	CLDN22	Claudin-22	Homo sapiens
Q96B33	Reviewed	CLDN23	Claudin-23	Homo sapiens
A6NM45	Reviewed	CLDN24/CLDN21	Putative claudin-24 (Claudin-21)	Homo sapiens
C9JDP6	Reviewed	CLDN25	Putative claudin-25	Homo sapiens
O15551	Reviewed	CLDN3	Claudin-3 (CPE-receptor 2)	Homo sapiens
H7C241	Reviewed	CLDN34	Claudin-34	Homo sapiens
O14493	Reviewed	CLDN4	Claudin-4 (CPE-receptor)	Homo sapiens
O00501	Reviewed	CLDN5	Claudin-5 (Transmembrane protein deleted in VCFS) (TMDVCF)	Homo sapiens
P56747	Reviewed	CLDN6	Claudin-6 (Skullin)	Homo sapiens
O95471	Reviewed	CLDN7	Claudin-7	Homo sapiens
P56748	Reviewed	CLDN8	Claudin-8	Homo sapiens
O95484	Reviewed	CLDN9	Claudin-9	Homo sapiens

### Expression of Claudins Is Dysregulated in Various Cancers

**FIGURE 2:**
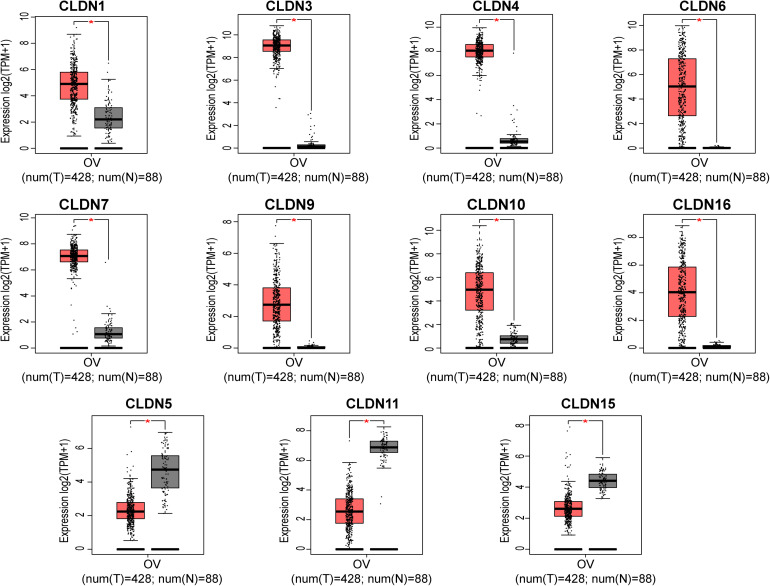
The mRNA expression of claudins in TCGA samples and the GTEx normal samples via GEPIA2. (*p < 0.01).

To explore the mRNA expression of the claudin family, we investigated the expression profiles of claudins in various cancers via the ONCOMINE database. The thresholds were: *p*-value of 0.05, fold change of 1.5, and gene rank of all. Significant analyses are shown in Supplementary Figure 1 (those with < 3 significant analyses were not considered). Results showed that most claudins were dysregulated in various cancers. To verify the expression of claudins in ovarian cancer, GEPIA2 was used to analyze mRNA expression in TCGA and GTEx samples. The | Log_2_FC| cutoff was set to 1.5 and the *p*-value cutoff was set to 0.05. As shown in Figure 2, eight genes were overexpressed in ovarian cancer samples compared with normal tissue samples and included CLDN1, CLDN3, CLDN4, CLDN6, CLDN7, CLDN9, CLDN10, and CLDN16. Furthermore, three genes showed low expression in the ovarian cancer samples compared with normal tissue samples and included CLDN5, CLDN11, and CLDN15.

### Correlation of Claudin Expression With Ovarian Cancer Prognosis

**FIGURE 3:**
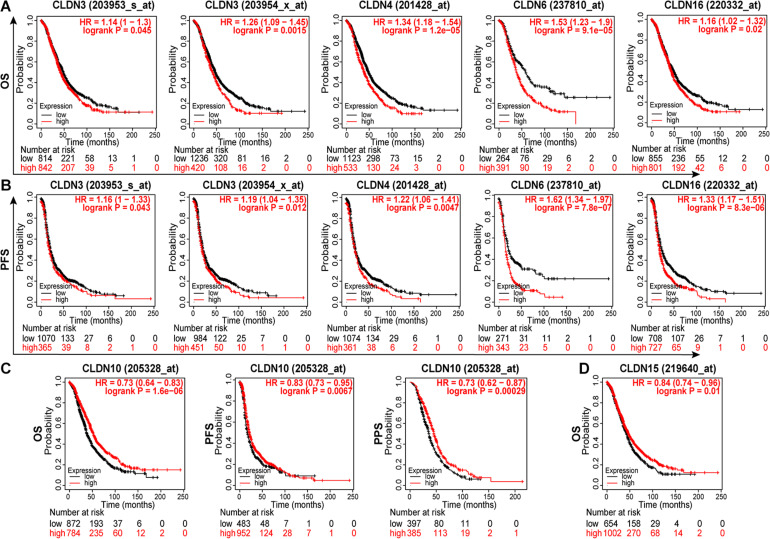
The relationship between claudin expression and the prognosis of ovarian cancer patients through Kaplan-Meier plotter. The overexpression of CLDN3, CLDN4, CLDN6, and CLDN16 were significantly correlated with poor OS **(A)** and PFS **(B)**. **(C)** The overexpression of CLDN10 predicted good OS, PFS, and PPS. **(D)** The low expression of CLDN15 predicted poor OS in ovarian cancer. OS, overall survival; PFS, progression free survival; PPS, post progression survival.

To identify genes with clinical significance, we studied the relationship between differentially expressed genes (DEGs) and ovarian cancer patient prognosis using the Kaplan-Meier plotter. As shown in Figure 3, overexpressed genes CLDN3, CLDN4, CLDN6, and CLDN16 were significantly correlated with poor OS (Figure 3A) and progression free survival (PFS) (Figure 3B). In addition, high expression of CLDN10 and CLDN15 were predictive of a good prognosis in ovarian cancer patients (Figures 3C,D). Surprisingly, CLDN10 was overexpressed in cancer, but patients with high expression of CLDN10 showed good OS (HR = 0.73, logrank *P* = 1.6e-06), PFS (HR = 0.83, logrank *P* = 0.0067), and post progression survival (PPS, HR = 0.73, logrank *P* = 0.00029). These results are somewhat counterintuitive, and the underlying mechanism requires further exploration.

TCGA projects have identified four molecular subtypes of high-grade serous ovarian carcinoma (HGSOC) (Cancer Genome Atlas Research Network, 2011): (i) the differentiated subtype; (ii) the immunoreactive subtype; (iii) the mesenchymal subtype; and (iv) the proliferative subtype. Among them, T-cell chemokine ligands, CXCL11 and CXCL10, and the receptor, CXCR3, characterized the immunoreactive subtype. Then, Thorsson et al. (2018) developed a global immune classification of solid tumors based on the transcriptomic profiles of 33 cancer types. They identified six distinct immune subtypes: C1 (Wound healing); C2 (IFN-γ dominant); C3 (Inflammatory); C4 (Lymphocyte depleted); C5 (Immunologically quiet); C6 (TGF-β dominant). These six categories represent features of the tumor microenvironment (Charoentong et al., 2017). In this research, we explored the relationships between the expression of differentially expressed genes related to prognosis and molecular subtypes or immune subtypes of ovarian cancer via the TISIDB (Ru et al., 2019). The Kruskal-Wallis test was used. As Supplementary Figure 2 shows, claudins including CLDN3, CLDN6, CLDN10, and CLDN15 are differentially expressed in different immune subtypes. And, claudins including CLDN3, CLDN4, CLDN6, CLDN10, and CLDN16 are differentially expressed in different molecular subtypes (Supplementary Figure 3). Among them, CLDN6 is relatively low expression, and CLDN10 is relatively high expression in the immunoreactive subtype.

### GSEA of Immunological Signature Gene Sets

**FIGURE 4:**
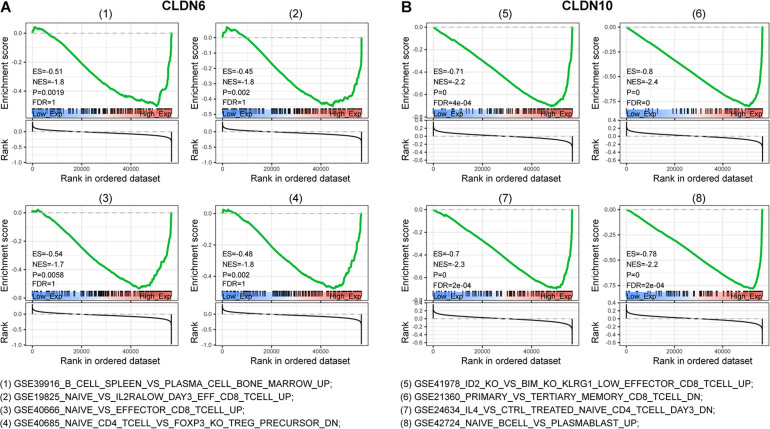
Gene set enrichment analysis (GSEA) of c7 (immunologic signatures) for CLDN6 and CLDN10. CLDN6 **(A)** and CLDN10 **(B)** were related to effector differentiation of B cell, CD8 T cell, and CD4 T cell.

To characterize the potential function of claudins, GSEA was performed using gene expression data from TCGA ovarian cancer patients. Immunological signature gene sets were used. As shown in Figure 4, CLDN6 and CLDN10 were related to the effector differentiation of B cell, CD4 T cell, and CD8 T cell.

### Correlation Analyses Between Claudins and Tumor Immune Microenvironment

**FIGURE 5:**
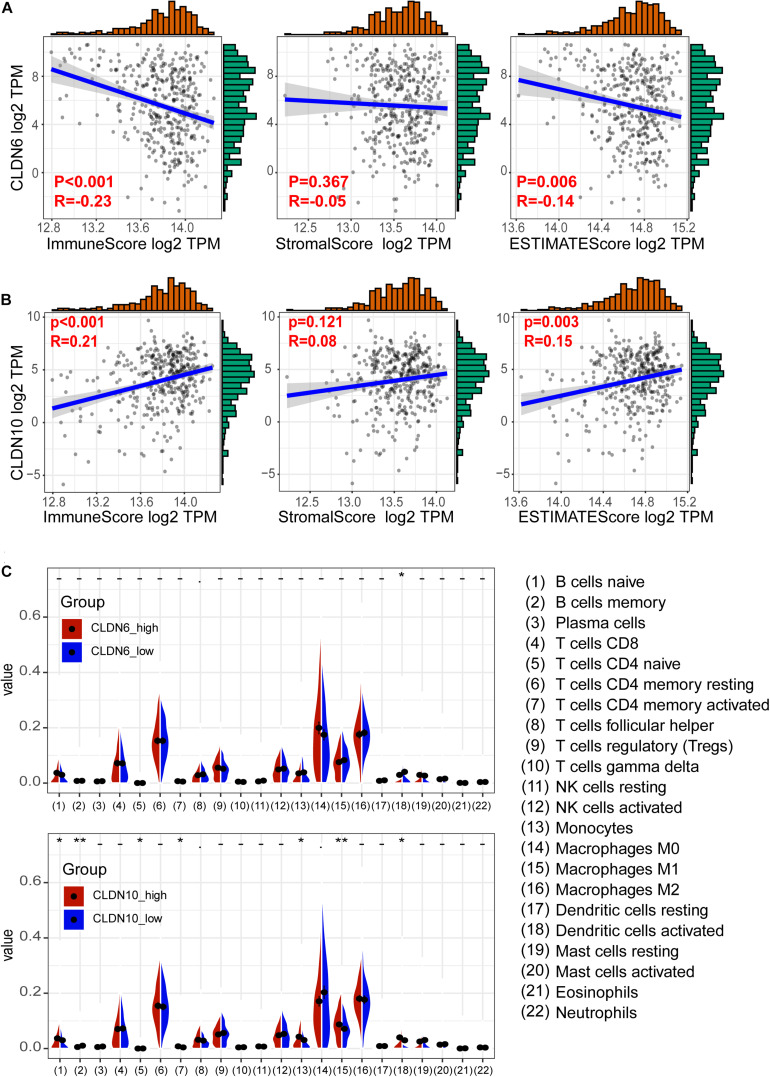
Relationship between claudins expression and tumor immune microenvironment. **(A)** The expression of CLDN6 has a negative correlation with immune score and ESTIMATE score. **(B)** The expression of CLDN10 has a positive correlation with immune score and ESTIMATE score. **(C)** The difference of 22 immune cells between the claudin-high group and claudin-low group (*p < 0.05, **p < 0.01).

To understand the role of claudins in immunity, we downloaded 379 RNA-seq FPKM (Fragments per kilobase per million) data of ovarian cancer from TCGA. Subsequently, the FPKM was converted to TPM (transcripts per million) (Li et al., 2010). The ESTIMATE algorithm (Yoshihara et al., 2013) was used to predict tumor purity based on TCGA ovarian cancer samples. Then, the relationship between claudin expression and the immune microenvironment was explored. As shown in Figure 5A, a significant negative correlation between CLDN6 expression and the immune score was observed (Spearman correlation = −0.23, *p* < 0.001). A significant positive correlation between CLDN10 expression and immune score (spearman correlation = 0.21, *p* < 0.001) was observed (Figure 5B). However, the expression levels of CLDN6 and CLDN10 were not correlated with the stromal score.

We next examined the relationship between immune cell infiltration and claudin expression. RNA-seq TPM data (*n* = 379) from TCGA ovarian cancer were used to assess 22 immune cells subtype concentrations with the CIBERSORT algorithm (Newman et al., 2019). TCGA samples were grouped by the median values of CLDN6 and CLDN10, respectively. Activated dendritic cells differed significantly between the CLDN6_high and CLDN6_low groups. Several cell types were significantly different between the CLDN10_high and CLDN10_low group, including naïve B cells, memory B cells, naïve CD4 T cells, CD4 memory-activated T cells, monocytes, M1 macrophages, and activated dendritic cells (Figure 5C).

**FIGURE 6:**
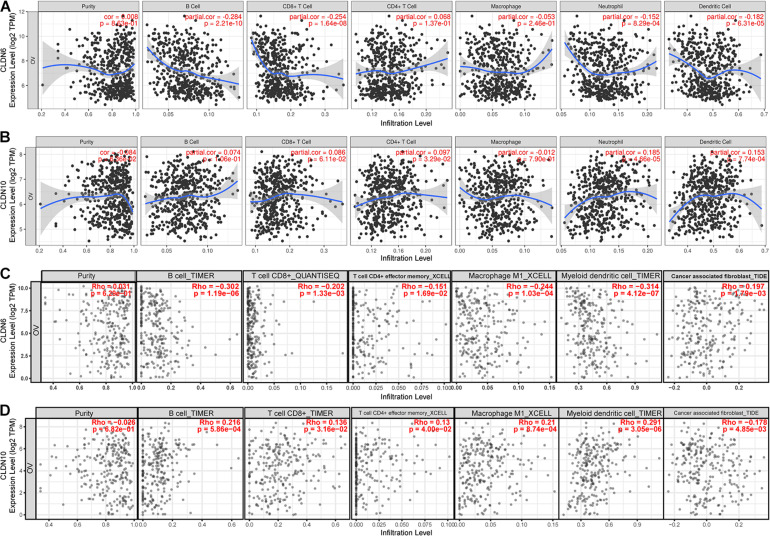
The relationship between immune cell infiltration and claudin expression. Correlation analysis of immune cell infiltration and CLDN6 expression **(A)**, and CLDN10 expression **(B)** based on the microarray expression values of ovarian cancer through TIMER. Correlation analysis of immune cell infiltration and CLDN6 expression **(C)**, and CLDN10 expression **(D)** based on available TCGA RNA-seq data of ovarian cancer via TIMER2.

The microarray expression values of ovarian cancer were used to calculate the abundances of six immune infiltrates (B cells, CD4^+^ T cells, CD8^+^ T cells, Neutrophils, Macrophages, and Dendritic cells) via the TIMER algorithm (Yoshihara et al., 2013). The gene expression levels correlated with tumor purity are displayed in the left-most panel (Figures 6A,B). Results showed that CLDN6 expression was negatively correlated with infiltration of B cell (partial correlation = −0.284, *p* = 2.21e-10), CD8^+^ T cells (partial correlation = −0.254, *p* = 1.64e-08), neutrophils (partial correlation = −0.152, *p* = 8.29e-04), and dendritic cells (partial correlation = −0.182, *p* = 6.31e-05) (Figure 6A). In contrast, there was a small but significant positive correlation between CLDN10 expression and infiltration of neutrophils (partial correlation = 0.185, *p* = 4.66e-05), and dendritic cells (partial correlation = 0.153, *p* = 7.74e-04) (Figure 6B).

To more accurately describe the relationship between gene expression and immune cell infiltration, we used the TIMER, CIBERSORT, quanTIseq, xCell, MCP-counter, and EPIC algorithms to assess the immune infiltration in tumor tissue (Sturm et al., 2019). TIMER2 provides a platform to analyze immune infiltrates across diverse cancer types based on available TCGA RNA-seq data (Li et al., 2016; Li T. et al., 2020). The correlations between claudin expression (CLDN6 and CLDN10) and immune cell infiltration in ovarian cancer are shown in Table 2. As seen in Figure 6C, CLDN6 was negatively correlated with immune cell infiltration, including that of B cells, CD8^+^ T cells, effector memory CD4^+^ T cells, M1 macrophages, and myeloid dendritic cells. In contrast, CLDN10 was positively correlated with immune cell infiltration, including that of B cells, CD8^+^ T cells, effector memory CD4^+^ T cells, M1 macrophages, and myeloid dendritic cells (Figure 6D). Relevant evidence suggests that cancer-associated fibroblasts (CAFs) play an important role in the progression of ovarian cancer (Mhawech-Fauceglia et al., 2014; Leung et al., 2018). Interestingly, here, CAFs also showed a positive correlation with CLDN6 expression, but a negative correlation with CLDN10 expression. In ovarian cancer, increased infiltration of tumor-infiltrating lymphocytes (TILs), and more specifically CD8^+^ T cells, have been proven to be associated with improved clinical outcomes (Sato et al., 2005; Hamanishi et al., 2007; Ovarian Tumor Tissue Analysis et al., 2017). These results suggest that CLDN6 and CLDN10 may participate in immune cell infiltration in ovarian cancer, and these mechanisms may be the reason for poor prognosis.

**TABLE 2:** Correlation analysis between claudins and immune infiltration in ovarian cancer via TIMER2.0.

		**CLDN6**			**CLDN10**		
**Cancer**	**Infiltrates**	**rho**	***p***	**adj.p**	**rho**	***p***	**adj.p**
OV (*n* = 303)	B cell memory_CIBERSORT	−0.018	0.777	0.9214	−0.1938	**	*
OV (*n* = 303)	B cell memory_CIBERSORT-ABS	−0.0185	0.7713	0.9214	−0.1795	**	*
OV (*n* = 303)	B cell memory_XCELL	−0.0386	0.5446	0.7855	0.091	0.1521	0.3381
OV (*n* = 303)	B cell naive_CIBERSORT	0.0053	0.9343	0.9895	0.255	***	***
OV (*n* = 303)	B cell naive_CIBERSORT-ABS	−0.0058	0.9272	0.9895	0.2577	***	***
OV (*n* = 303)	B cell naive_XCELL	0.0915	0.15	0.4803	−0.142	*	0.0952
OV (*n* = 303)	B cell plasma_CIBERSORT	0.1164	0.0666	0.3075	−0.0337	0.5963	0.7755
OV (*n* = 303)	B cell plasma_CIBERSORT-ABS	0.0741	0.2443	0.5768	0.0036	0.9552	0.9837
OV (*n* = 303)	B cell plasma_XCELL	0.04	0.5302	0.7759	−0.12	0.0587	0.1821
OV (*n* = 303)	B cell_EPIC	0.045	0.4801	0.7541	−0.149	*	0.0782
OV (*n* = 303)	B cell_MCPCOUNTER	0.2482	***	**	−0.0836	0.1888	0.3814
OV (*n* = 303)	B cell_QUANTISEQ	0.1153	0.0694	0.3139	−0.1177	0.0636	0.1866
OV (*n* = 303)	B cell_TIMER	−0.3021	***	***	0.2164	***	**
OV (*n* = 303)	B cell_XCELL	−0.1283	*	0.2616	0.0756	0.2345	0.4401
OV (*n* = 303)	Cancer associated fibroblast_EPIC	0.1377	*	0.1353	−0.0907	0.1537	0.4081
OV (*n* = 303)	Cancer associated fibroblast_MCPCOUNTER	0.1594	*	0.0746	−0.0955	0.133	0.3766
OV (*n* = 303)	Cancer associated fibroblast_TIDE	0.197	**	*	−0.178	**	*
OV (*n* = 303)	Cancer associated fibroblast_XCELL	0.1913	**	*	−0.1201	0.0585	0.2122
OV (*n* = 303)	Class-switched memory B cell_XCELL	−0.1073	0.091	0.3747	0.1094	0.085	0.2267
OV (*n* = 303)	Common lymphoid progenitor_XCELL	−0.0628	0.3235	0.6596	0.0795	0.2112	0.4607
OV (*n* = 303)	Common myeloid progenitor_XCELL	−0.1444	*	0.139	0.0333	0.6009	0.8165
OV (*n* = 303)	Endothelial cell_EPIC	0.092	0.1478	0.4554	−0.1135	0.0738	0.2627
OV (*n* = 303)	Endothelial cell_MCPCOUNTER	0.15	*	0.1218	−0.1109	0.0807	0.2771
OV (*n* = 303)	Endothelial cell_XCELL	0.0923	0.1466	0.4554	−0.0893	0.16	0.403
OV (*n* = 303)	Eosinophil_CIBERSORT	0.1312	*	0.1921	−0.006	0.9255	0.9687
OV (*n* = 303)	Eosinophil_CIBERSORT-ABS	0.1299	*	0.1983	−0.0054	0.9323	0.9707
OV (*n* = 303)	Eosinophil_XCELL	0.0472	0.4588	0.7698	−0.0908	0.1531	0.3919
OV (*n* = 303)	Granulocyte-monocyte progenitor_XCELL	0.0423	0.5061	0.7873	0.0061	0.9236	0.9687
OV (*n* = 303)	Hematopoietic stem cell_XCELL	0.0704	0.2685	0.6192	−0.1648	**	0.0568
OV (*n* = 303)	Macrophage M0_CIBERSORT	0.12	0.0586	0.2045	−0.1693	**	*
OV (*n* = 303)	Macrophage M0_CIBERSORT-ABS	0.0854	0.1791	0.431	−0.1219	0.0546	0.168
OV (*n* = 303)	Macrophage M1_CIBERSORT	−0.1565	*	0.0812	0.1868	**	*
OV (*n* = 303)	Macrophage M1_CIBERSORT-ABS	−0.1201	0.0585	0.2045	0.1764	**	*
OV (*n* = 303)	Macrophage M1_QUANTISEQ	−0.1115	0.0792	0.2541	0.1631	**	*
OV (*n* = 303)	Macrophage M1_XCELL	−0.2436	***	**	0.2096	***	**
OV (*n* = 303)	Macrophage M2_CIBERSORT	−0.1332	*	0.1481	0.0946	0.1366	0.3176
OV (*n* = 303)	Macrophage M2_CIBERSORT-ABS	−0.1201	0.0585	0.2045	0.1292	*	0.1388
OV (*n* = 303)	Macrophage M2_QUANTISEQ	−0.0632	0.3207	0.6029	0.1233	0.0521	0.1619
OV (*n* = 303)	Macrophage M2_TIDE	0.3074	***	***	−0.2819	***	***
OV (*n* = 303)	Macrophage M2_XCELL	−0.2827	***	***	0.0992	0.1183	0.2886
OV (*n* = 303)	Macrophage/Monocyte_MCPCOUNTER	−0.1563	*	0.0812	0.0675	0.2884	0.5842
OV (*n* = 303)	Macrophage/Monocyte_MCPCOUNTER	−0.1563	*	0.1115	0.0675	0.2884	0.5244
OV (*n* = 303)	Macrophage_EPIC	−0.1983	**	*	0.1515	*	0.0698
OV (*n* = 303)	Macrophage_TIMER	0.0371	0.5602	0.7984	−0.1785	**	*
OV (*n* = 303)	Macrophage_XCELL	−0.2767	***	***	0.1879	**	*
OV (*n* = 303)	Mast cell activated_CIBERSORT	0.0135	0.8325	0.9299	−0.0271	0.6699	0.8355
OV (*n* = 303)	Mast cell activated_CIBERSORT-ABS	0.0118	0.8527	0.9352	−0.0284	0.6555	0.8323
OV (*n* = 303)	Mast cell resting_CIBERSORT	−0.0645	0.3106	0.65	0.0765	0.2289	0.4775
OV (*n* = 303)	Mast cell resting_CIBERSORT-ABS	−0.0775	0.223	0.5626	0.0979	0.1233	0.3433
OV (*n* = 303)	Mast cell_XCELL	−0.1516	*	0.1157	−0.0698	0.2723	0.5282
OV (*n* = 303)	MDSC_TIDE	0.3588	***	***	−0.1393	*	0.1339
OV (*n* = 303)	Monocyte_CIBERSORT	0.0449	0.481	0.7776	0.0739	0.2454	0.5578
OV (*n* = 303)	Monocyte_CIBERSORT-ABS	−0.0003	0.9966	0.9966	0.124	0.0507	0.2355
OV (*n* = 303)	Monocyte_MCPCOUNTER	−0.1563	*	0.1115	0.0675	0.2884	0.5842
OV (*n* = 303)	Monocyte_QUANTISEQ	−0.3974	***	***	0.1651	**	0.0626
OV (*n* = 303)	Monocyte_XCELL	−0.1109	0.0807	0.3318	0.0824	0.195	0.5043
OV (*n* = 303)	Myeloid dendritic cell activated_CIBERSORT	−0.1643	**	0.0559	0.1554	*	0.069
OV (*n* = 303)	Myeloid dendritic cell activated_CIBERSORT-ABS	−0.1626	*	0.0573	0.1618	*	0.0564
OV (*n* = 303)	Myeloid dendritic cell activated_XCELL	−0.2327	***	**	0.1691	**	*
OV (*n* = 303)	Myeloid dendritic cell resting_CIBERSORT	−0.0371	0.5605	0.7955	−0.0546	0.3908	0.635
OV (*n* = 303)	Myeloid dendritic cell resting_CIBERSORT-ABS	−0.0367	0.5642	0.7962	−0.0475	0.4551	0.6843
OV (*n* = 303)	Myeloid dendritic cell_MCPCOUNTER	−0.1032	0.1044	0.2989	0.0276	0.6652	0.8057
OV (*n* = 303)	Myeloid dendritic cell_QUANTISEQ	0.363	***	***	−0.1552	*	0.0693
OV (*n* = 303)	Myeloid dendritic cell_TIMER	−0.3143	***	***	0.2908	***	***
OV (*n* = 303)	Myeloid dendritic cell_XCELL	−0.1196	0.0595	0.2138	0.1565	*	0.0675
OV (*n* = 303)	Neutrophil_CIBERSORT	−0.1029	0.1053	0.4127	0.1114	0.0793	0.2453
OV (*n* = 303)	Neutrophil_CIBERSORT-ABS	−0.0951	0.1345	0.4605	0.1072	0.0913	0.2681
OV (*n* = 303)	Neutrophil_MCPCOUNTER	−0.0017	0.9786	0.9929	−0.0367	0.5639	0.7514
OV (*n* = 303)	Neutrophil_QUANTISEQ	0.1785	**	0.0595	−0.0207	0.7447	0.863
OV (*n* = 303)	Neutrophil_TIMER	−0.0724	0.2552	0.61	0.0614	0.3348	0.5858
OV (*n* = 303)	Neutrophil_XCELL	−0.0869	0.1714	0.5122	0.0842	0.1851	0.418
OV (*n* = 303)	NK cell activated_CIBERSORT	−0.0263	0.6796	0.8663	0.0296	0.6423	0.8424
OV (*n* = 303)	NK cell activated_CIBERSORT-ABS	−0.0404	0.5256	0.7786	0.12	0.0587	0.2122
OV (*n* = 303)	NK cell resting_CIBERSORT	−0.1009	0.1124	0.3225	−0.0246	0.6989	0.8788
OV (*n* = 303)	NK cell resting_CIBERSORT-ABS	−0.1109	0.0808	0.266	−0.0226	0.7224	0.8908
OV (*n* = 303)	NK cell_EPIC	−0.1815	**	*	0.1149	0.0703	0.2474
OV (*n* = 303)	NK cell_MCPCOUNTER	−0.1553	*	0.0848	0.1402	*	0.12
OV (*n* = 303)	NK cell_QUANTISEQ	−0.0556	0.3821	0.6781	0.0411	0.519	0.7789
OV (*n* = 303)	NK cell_XCELL	−0.0824	0.1951	0.4491	0.0799	0.2087	0.4765
OV (*n* = 303)	Plasmacytoid dendritic cell_XCELL	−0.208	***	*	0.2213	***	**
OV (*n* = 303)	T cell CD4^+^ (non-regulatory)_QUANTISEQ	−0.0536	0.3998	0.7259	−0.0638	0.3156	0.5912
OV (*n* = 303)	T cell CD4^+^ (non-regulatory)_XCELL	0.0077	0.9032	0.9663	−0.0723	0.2555	0.5347
OV (*n* = 303)	T cell CD4^+^ central memory_XCELL	0.0456	0.4736	0.7811	0.0344	0.5892	0.8122
OV (*n* = 303)	T cell CD4^+^ effector memory_XCELL	−0.1513	*	0.1109	0.1302	*	0.1625
OV (*n* = 303)	T cell CD4^+^ memory activated_CIBERSORT	−0.0047	0.9411	0.9798	0.0538	0.3982	0.6743
OV (*n* = 303)	T cell CD4^+^ memory activated_CIBERSORT-ABS	−0.0041	0.9485	0.9798	0.0526	0.409	0.6835
OV (*n* = 303)	T cell CD4^+^ memory resting_CIBERSORT	0.1047	0.0994	0.329	0.015	0.8141	0.9242
OV (*n* = 303)	T cell CD4^+^ memory resting_CIBERSORT-ABS	0.0014	0.9827	0.992	0.0943	0.1378	0.3757
OV (*n* = 303)	T cell CD4^+^ memory_XCELL	0.0253	0.6916	0.897	0.0693	0.2762	0.5595
OV (*n* = 303)	T cell CD4^+^ naive_CIBERSORT	0.1349	*	0.1741	−0.1428	*	0.1147
OV (*n* = 303)	T cell CD4^+^ naive_CIBERSORT-ABS	0.1349	*	0.1741	−0.1428	*	0.1147
OV (*n* = 303)	T cell CD4^+^ naive_XCELL	−0.1611	*	0.0828	0.1101	0.083	0.2652
OV (*n* = 303)	T cell CD4^+^ Th1_XCELL	−0.1385	*	0.1608	0.0499	0.4328	0.7009
OV (*n* = 303)	T cell CD4^+^ Th2_XCELL	0.0625	0.3263	0.6506	0.0766	0.2287	0.522
OV (*n* = 303)	T cell CD4^+^ _EPIC	0.0428	0.5014	0.8099	−0.0148	0.8168	0.9242
OV (*n* = 303)	T cell CD4^+^ _TIMER	0.1149	0.0703	0.2735	−0.0058	0.9273	0.9753
OV (*n* = 303)	T cell CD8^+^ central memory_XCELL	−0.1749	**	*	0.1568	*	0.0801
OV (*n* = 303)	T cell CD8^+^ effector memory_XCELL	0.0858	0.177	0.4688	0.0796	0.2107	0.4441
OV (*n* = 303)	T cell CD8^+^ naive_XCELL	0.1924	**	*	−0.1191	0.0606	0.2145
OV (*n* = 303)	T cell CD8^+^ _CIBERSORT	−0.0534	0.4012	0.6829	0.0301	0.6366	0.8318
OV (*n* = 303)	T cell CD8^+^ _CIBERSORT-ABS	−0.0453	0.4765	0.7086	0.0702	0.2695	0.5033
OV (*n* = 303)	T cell CD8^+^ _EPIC	0.0434	0.4951	0.7166	−0.0542	0.3944	0.6552
OV (*n* = 303)	T cell CD8^+^ _MCPCOUNTER	−0.0322	0.613	0.7909	0.0925	0.1455	0.3528
OV (*n* = 303)	T cell CD8^+^ _QUANTISEQ	−0.2023	**	*	0.1851	**	*
OV (*n* = 303)	T cell CD8^+^ _TIMER	−0.1707	**	*	0.1363	*	0.139
OV (*n* = 303)	T cell CD8^+^ _XCELL	−0.0544	0.3923	0.6765	−0.0078	0.9028	0.9629
OV (*n* = 303)	T cell follicular helper_CIBERSORT	−0.036	0.5716	0.8255	0.0032	0.9605	0.9889
OV (*n* = 303)	T cell follicular helper_CIBERSORT-ABS	−0.0618	0.3316	0.7046	0.058	0.3618	0.6466
OV (*n* = 303)	T cell gamma delta_CIBERSORT	−0.0281	0.6591	0.8771	−0.0738	0.2458	0.5578
OV (*n* = 303)	T cell gamma delta_CIBERSORT-ABS	−0.0276	0.6642	0.8771	−0.0735	0.2481	0.5578
OV (*n* = 303)	T cell gamma delta_XCELL	−0.0545	0.3918	0.7431	0.03	0.6374	0.8533
OV (*n* = 303)	T cell NK_XCELL	−0.1745	**	0.064	−0.001	0.9869	0.9937
OV (*n* = 303)	T cell regulatory (Tregs)_CIBERSORT	−0.0056	0.9299	0.9886	−0.0417	0.5123	0.7769
OV (*n* = 303)	T cell regulatory (Tregs)_CIBERSORT-ABS	−0.0278	0.6622	0.8546	−0.006	0.9248	0.971
OV (*n* = 303)	T cell regulatory (Tregs)_QUANTISEQ	−0.001	0.9873	0.9998	0.1678	**	*
OV (*n* = 303)	T cell regulatory (Tregs)_XCELL	0.0683	0.283	0.575	0.0402	0.5276	0.783

*P < 0.05;**P < 0.01;***P < 0.001.

### Relationship Between Claudin Expression and Gene Markers of Immune Cells

To further illustrate the correlation between claudins and the immune microenvironment, we analyzed the relationship between CLDN6 and CLDN10 expression and gene markers of various immune cells in ovarian cancer (TIMER2 database), including B cells, T cells (general), CD8^+^ T cells, macrophages, dendritic cells, neutrophils, monocytes, natural killer (NK) cells, and regulatory T cells (Tregs) (Table 3). Purity-adjusted correlation heatmaps are shown in Supplementary Figure 4. After correlation adjustment by purity, CLDN6 expression was negatively correlated with most gene markers of dendritic cells, M1 macrophages, monocytes, NK cells, and tumor-associated macrophages (TAMs) in ovarian cancer. In contrast, CLDN10 expression was positively correlated with gene markers of dendritic cells, T cells (general), and TAMs in ovarian cancer.

**TABLE 3:** Correlation analysis between claudins and markers of immune cells in ovarian cancer via TIMER2.0.

			**CLDN6**			**CLDN10**		
**Cancer**	**Immune cells**	**Gene markers**	**rho**	***p***	**adj.p**	**rho**	***p***	**adj.p**
OV (*n* = 303)	B cell	CD19	0.122	0.052	0.189	−0.075	0.268	0.477
OV (*n* = 303)	B cell	CD79A	0.022	0.692	0.853	−0.063	0.307	0.521
OV (*n* = 303)	CD8^+^ T cell	CD8A	−0.103	0.103	0.305	0.0977	0.121	0.298
OV (*n* = 303)	CD8^+^ T cell	CD8B	−0.032	0.613	0.798	0.0925	0.145	0.336
OV (*n* = 303)	DC	CD1C	−0.158	*	0.098	0.0864	0.172	0.467
OV (*n* = 303)	DC	HLA-DPA1	−0.253	***	**	0.2298	***	**
OV (*n* = 303)	DC	HLA-DPB1	−0.3	***	***	0.2535	***	***
OV (*n* = 303)	DC	HLA-DQB1	−0.224	***	**	0.2259	***	**
OV (*n* = 303)	DC	HLA-DRA	−0.325	***	***	0.2428	***	**
OV (*n* = 303)	DC	ITGAX	−0.182	**	*	0.0859	0.178	0.469
OV (*n* = 303)	DC	NRP1	0.1252	*	0.235	−0.004	0.996	0.997
OV (*n* = 303)	M1 Macrophage	IRF5	−0.186	**	*	0.0896	0.157	0.342
OV (*n* = 303)	M1 Macrophage	NOS2	0.1436	*	0.106	−0.033	0.545	0.757
OV (*n* = 303)	M1 Macrophage	PTGS2	0.0961	0.135	0.347	0.0093	0.886	0.942
OV (*n* = 303)	M2 Macrophage	CD163	−0.106	0.096	0.289	0.0646	0.31	0.529
OV (*n* = 303)	M2 Macrophage	MS4A4A	−0.112	0.072	0.238	0.1147	0.077	0.206
OV (*n* = 303)	M2 Macrophage	VSIG4	−0.152	*	0.086	0.0768	0.224	0.433
OV (*n* = 303)	Monocyte	CD86	−0.222	***	**	0.1457	*	0.084
OV (*n* = 303)	Monocyte	CSF1R	−0.196	**	*	0.0717	0.256	0.473
OV (*n* = 303)	NK cell	KIR2DL1	−0.001	0.924	0.986	0.0991	0.117	0.388
OV (*n* = 303)	NK cell	KIR2DL3	−0.226	***	**	0.1527	*	0.096
OV (*n* = 303)	NK cell	KIR2DL4	−0.258	***	**	0.1563	*	0.08
OV (*n* = 303)	NK cell	KIR2DS4	−0.097	0.121	0.391	0.0847	0.185	0.475
OV (*n* = 303)	NK cell	KIR3DL1	0.019	0.764	0.936	0.1037	0.105	0.348
OV (*n* = 303)	NK cell	KIR3DL2	−0.063	0.318	0.631	0.1495	*	0.107
OV (*n* = 303)	NK cell	KIR3DL3	−0.044	0.466	0.751	0.0571	0.368	0.682
OV (*n* = 303)	Neutrophil	CCR7	−0.068	0.324	0.633	0.0943	0.138	0.421
OV (*n* = 303)	Neutrophil	CEACAM8	−0.065	0.344	0.658	−0.034	0.619	0.839
OV (*n* = 303)	Neutrophil	ITGAM	−0.185	**	*	0.0575	0.367	0.682
OV (*n* = 303)	T cell (general)	CD2	−0.157	*	0.065	0.1651	**	*
OV (*n* = 303)	T cell (general)	CD3D	−0.142	*	0.106	0.1524	*	0.077
OV (*n* = 303)	T cell (general)	CD3E	−0.126	*	0.175	0.1581	*	0.051
OV (*n* = 303)	TAM	CCL2	−0.171	**	*	0.1709	**	*
OV (*n* = 303)	TAM	CD68	−0.203	**	*	0.105	0.093	0.258
OV (*n* = 303)	TAM	IL10	0.046	0.432	0.707	−0.007	0.948	0.979
OV (*n* = 303)	Tfh	IL21	−0.128	*	0.164	−0.016	0.844	0.934
OV (*n* = 303)	Tfh	BCL6	−0.195	**	*	0.1285	*	0.158
OV (*n* = 303)	Th1	IFNG	−0.088	0.186	0.438	0.1323	*	0.146
OV (*n* = 303)	Th1	STAT1	−0.077	0.229	0.489	0.0894	0.158	0.384
OV (*n* = 303)	Th1	STAT4	−0.009	0.873	0.959	0.0768	0.225	0.468
OV (*n* = 303)	Th1	TBX21	−0.159	*	0.078	0.1587	*	0.063
OV (*n* = 303)	Th1	TNF	−0.038	0.568	0.778	0.02	0.759	0.882
OV (*n* = 303)	Th17	IL17A	−0.073	0.265	0.528	0.0043	0.943	0.981
OV (*n* = 303)	Th17	STAT3	−0.042	0.488	0.731	0.0117	0.857	0.938
OV (*n* = 303)	Th2	GATA3	−0.061	0.305	0.562	−0.084	0.206	0.436
OV (*n* = 303)	Th2	IL13	−0.017	0.761	0.893	0.0647	0.303	0.575
OV (*n* = 303)	Th2	STAT5A	−0.115	0.062	0.215	−0.051	0.423	0.681
OV (*n* = 303)	Th2	STAT6	−0.046	0.458	0.711	0.0869	0.176	0.396
OV (*n* = 303)	Treg	CCR8	−0.004	0.893	0.968	0.0211	0.741	0.882
OV (*n* = 303)	Treg	FOXP3	−0.059	0.415	0.675	0.0635	0.317	0.588
OV (*n* = 303)	Treg	STAT5B	0.152	*	0.081	−0.1677	**	*
OV (*n* = 303)	Treg	TGFB1	−0.127	0.052	0.181	0.0153	0.815	0.924

DC, Dendritic cell; NK cel, Natural killer cell; TAM, Tumor-associated macrophage; Tfh, Follicular helper T cell; Treg, Regulatory T cell; *P < 0.05; **P < 0.01; ***P < 0.001.

Studies have shown that the tumor-infiltrating immune cells mentioned above are related to the tumor immunotherapy response (Rodriguez et al., 2018). Immune cell-based immunotherapy (Baci et al., 2020), including NK Cells (Nersesian et al., 2019) and dendritic cells (Stiff et al., 2013), play important roles in the treatment of ovarian cancer. Taken together, these analyses and our research indicate that CLDN6 and CLDN10 may play important roles in immunotherapy in the future.

## Discussion

CLDN6 and CLDN10 are important components of the claudin family related to tight junctions. Claudins were considered promising targets for diagnosis and therapy since they were involved in uncontrolled cancer growth and metastasis (Martin and Jiang, 2001; Morin, 2005; Bose and Mukhopadhyay, 2010). Moreover, studies have shown that they not only play a vital role in tumorigenesis (Swisshelm et al., 2005; Arabzadeh et al., 2007; Hagen, 2019), but also drug resistance (Gao et al., 2017).

CLDN6 had been demonstrated abnormal expression and can be a prognostic marker in cancers including ovarian cancer (Wang et al., 2013), endometrial cancer (Kojima et al., 2020), gastric cancer (Kohmoto et al., 2020), breast carcinoma (Liu et al., 2016; Jia et al., 2019), and lung cancer (Micke et al., 2014). Bioinformatic analysis has revealed that CLDN6 is regulated by a diverse set of transcription factors and promotes cancer cell behavior via the ASK1-p38/JNK MAPK secretory signaling pathway (Lin et al., 2017). A study revealed that CLDN6 may be a novel targeted therapy for ovarian cancer as a receptor for clostridium perfringens enterotoxin (Lal-Nag et al., 2012). In addition, 6PHU3, a T-cell-engaging bispecific single chain antibody with anti-CD3/anti-CLDN6 specificities, upregulated the cytotoxicity of T cells and made T cells acquire an effector phenotype (Stadler et al., 2016). Another recent study showed that CLDN6 as a chimeric antigen receptor target in solid tumors can be a strategy to overcome inefficient CAR-T cell stimulation *in vivo* (Reinhard et al., 2020). These studies suggested that CLDN6 has important research value in the treatment of cancer.

CLDN10, a glandular epithelial marker in epithelial ovarian cancer (Seo et al., 2010), was reported to be a key immune-related gene in clear cell renal cell carcinoma (Yang et al., 2021) and papillary thyroid carcinoma (Xiang et al., 2020). Furthermore, CLDN10 expression has proved to be a prognostic marker for ovarian cancer (Li Z. et al., 2020).

The present study combined and analyzed the prognostic potential of CLDN6 and CLDN10 with the tumor immune microenvironment. Consistent with previous reports, both CLDN6 and CLDN10 showed high expression in ovarian cancer. Prognostic analysis showed that the overexpression of CLDN6 was related to a poor prognosis for patients with ovarian cancer. However, CLDN10 overexpression predicted a better prognosis compared to the low CLDN10 expression group. We also found that CLDN6 overexpression was negatively related to immune cell infiltration, whereas CLDN10 overexpression was positively correlated with immune cell infiltration. Moreover, we found that CLDN6 and CLDN10 were related to gene markers of dendritic cells, NK cells, and TAMs. These results may explain why the overexpression of CLDN6 and low expression of CLDN10 predict poor OS in ovarian cancer. This study revealed that the prognostic potential of CLDN6 and CLDN10 is related to the tumor immune microenvironment in ovarian cancer.

Relevant evidence has emerged that immune-related gene expression and TILs are related to the prognosis, recurrence (Ojalvo et al., 2018), and chemotherapeutic response (Choi et al., 2020) of ovarian cancer. Furthermore, the presence of TILs may improve clinical outcomes in ovarian cancer patients (Odunsi, 2017). Immune cell-based immunotherapy (Baci et al., 2020), including NK Cells (Nersesian et al., 2019) and dendritic cells (Stiff et al., 2013), play an important role in the treatment of ovarian cancer. Previous studies and our analyses suggest that CLDN6 may be involved in immune evasion and that they could be an ideal candidate for immunotherapy in ovarian cancer. Future studies on the combined application of claudin-based molecular targeted therapy and immunotherapy are necessary.

## Conclusion

CLDN6 and CLDN10 were identified as potential prognostic biomarkers and were correlated with immune cell infiltration in ovarian cancer. Our results revealed new roles for CLDN6 and CLDN10 in ovarian cancer and their potential as therapeutic targets in cancer treatment.

## Data Availability Statement

The original contributions presented in the study are included in the article/Supplementary Material, further inquiries can be directed to the corresponding author/s.

## Author Contributions

PeW was responsible for the study conception and design. PG, TP, CC, and SL were involved in data acquisition, data analysis, and interpretation. PG drafted the manuscript and took charge of supervising the manuscript. All authors read and approved the manuscript.

## Conflict of Interest

The authors declare that the research was conducted in the absence of any commercial or financial relationships that could be construed as a potential conflict of interest.

## Footnotes

1 https://www.cbioportal.org/

2 https://www.ONCOMINE.org/resource/login.html

3 http://gepia2.cancer-pku.cn/

4 http://kmplot.com/analysis/index.php?p=background

5 https://cistrome.shinyapps.io/timer/

6 https://www.uniprot.org/

## Supplementary Material

Supplementary Figure 1 The significant unique analyses of claudins expression in ONCOMINE. (Red: overexpression; Blue: low expression).

Supplementary Figure 2 Associations between the expression of claudins and immune subtypes of ovarian cancer.

Supplementary Figure 3 Associations between the expression of claudins and molecular subtypes of ovarian cancer.

Supplementary Figure 4 The correlations between claudins (CLDN6 and CLDN10) expression and gene markers of immune cells across gynecologic oncology (Red: positive correlation; Blue: negative correlation).

## Abbreviations

GEPIA: Gene Expression Profiling Interactive Analysis
TIMER: Tumor Immune Estimation Resource
GSEA: gene set enrichment analyses
TCGA: The Cancer Genome Atlas
GEO: Gene Expression Omnibus
EGA: European Genome-Phenome Archive
FPKM: Fragments per kilobase per million
TPM: transcripts per million
TILs: tumor-infiltrating lymphocytes
NK: natural killer cells
Tregs: regulatory T cells
CAFs: cancer associated fibroblasts
TAMs: Tumor-associated macrophages
CPE: clostridium perfringens enterotoxin
OS: overall survival
PFS: progression free survival
PPS: post progression survival.
