# Supplementary figures and images for: Association of CLDN6 and CLDN10 With Immune Microenvironment in Ovarian Cancer: A Study of the Claudin Family
Source: Front Genet. 2021 Jun 23;12:595436. doi: 10.3389/fgene.2021.595436 (PMC8262617; doi:10.3389/fgene.2021.595436)

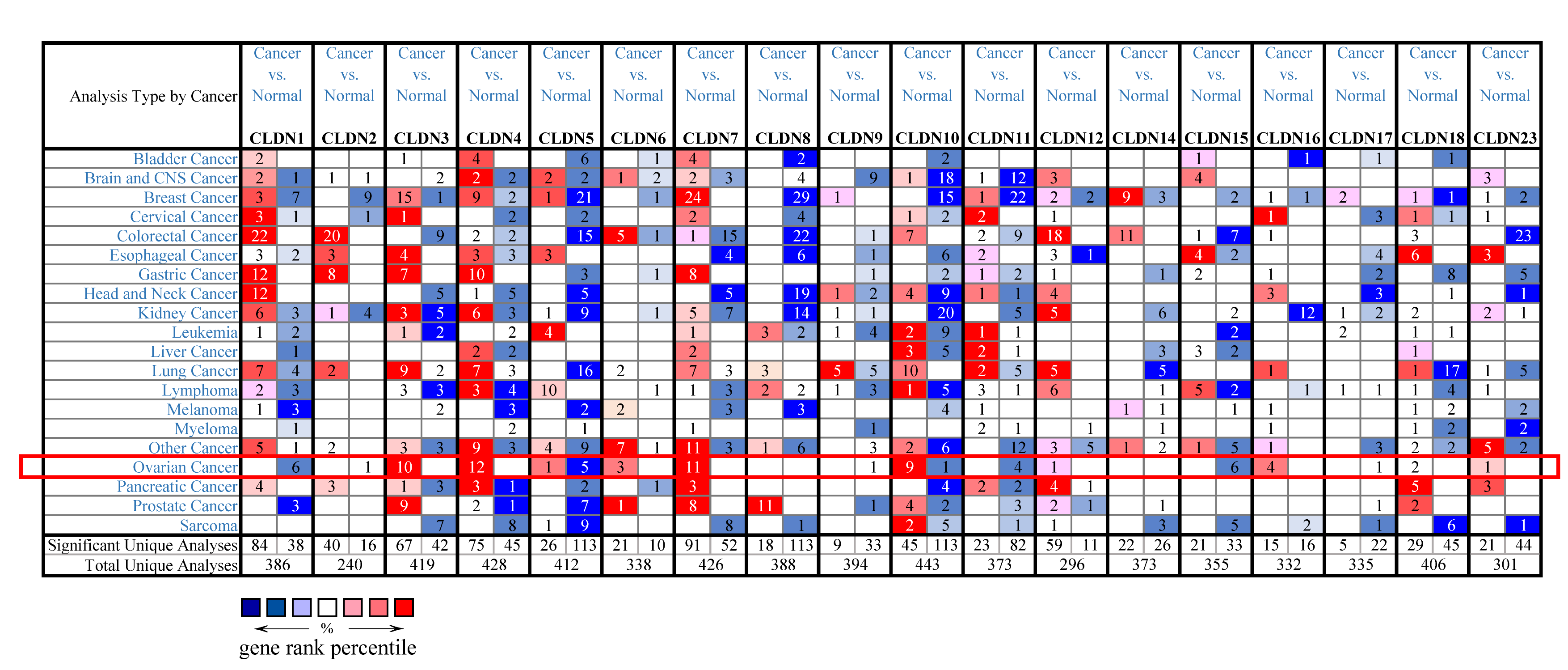

Supplement: Supplementary Figure 1 — The significant unique analyses of claudins expression in ONCOMINE. (Red: overexpression; Blue: low expression). [file Image_1.TIF]

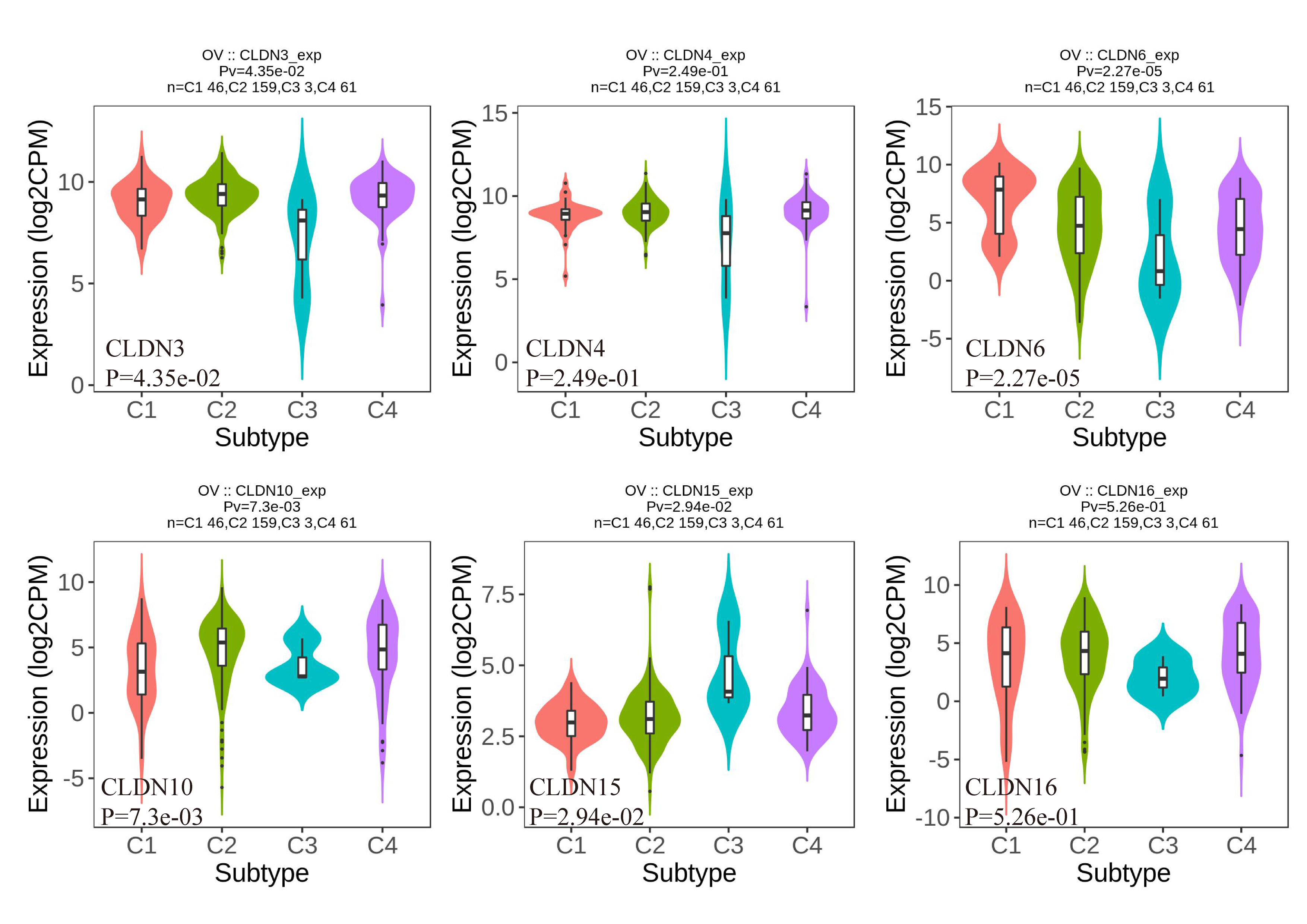

Supplement: Supplementary Figure 2 — Associations between the expression of claudins and immune subtypes of ovarian cancer. [file Image_2.TIF]

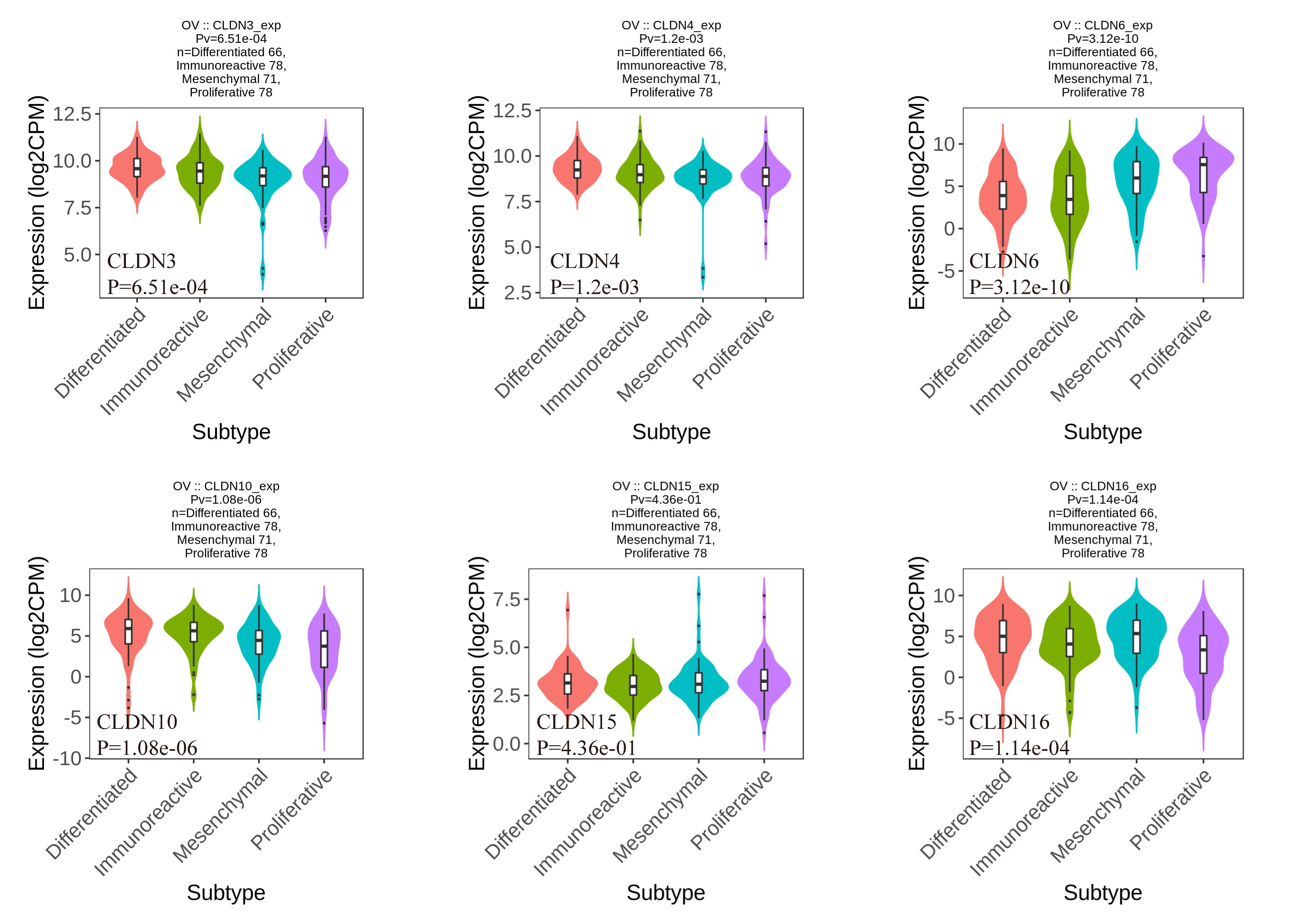

Supplement: Supplementary Figure 3 — Associations between the expression of claudins and molecular subtypes of ovarian cancer. [file Image_3.TIF]

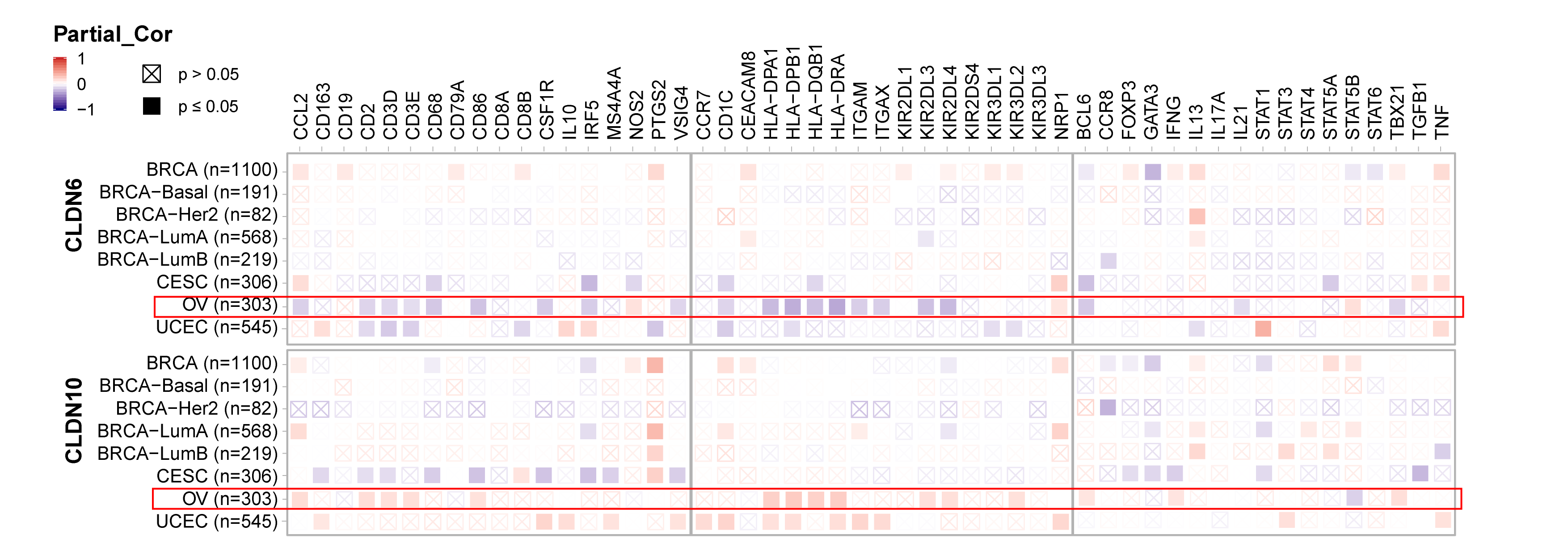

Supplement: Supplementary Figure 4 — The correlations between claudins (CLDN6 and CLDN10) expression and gene markers of immune cells across gynecologic oncology (Red: positive correlation; Blue: negative correlation). [file Image_4.TIF]

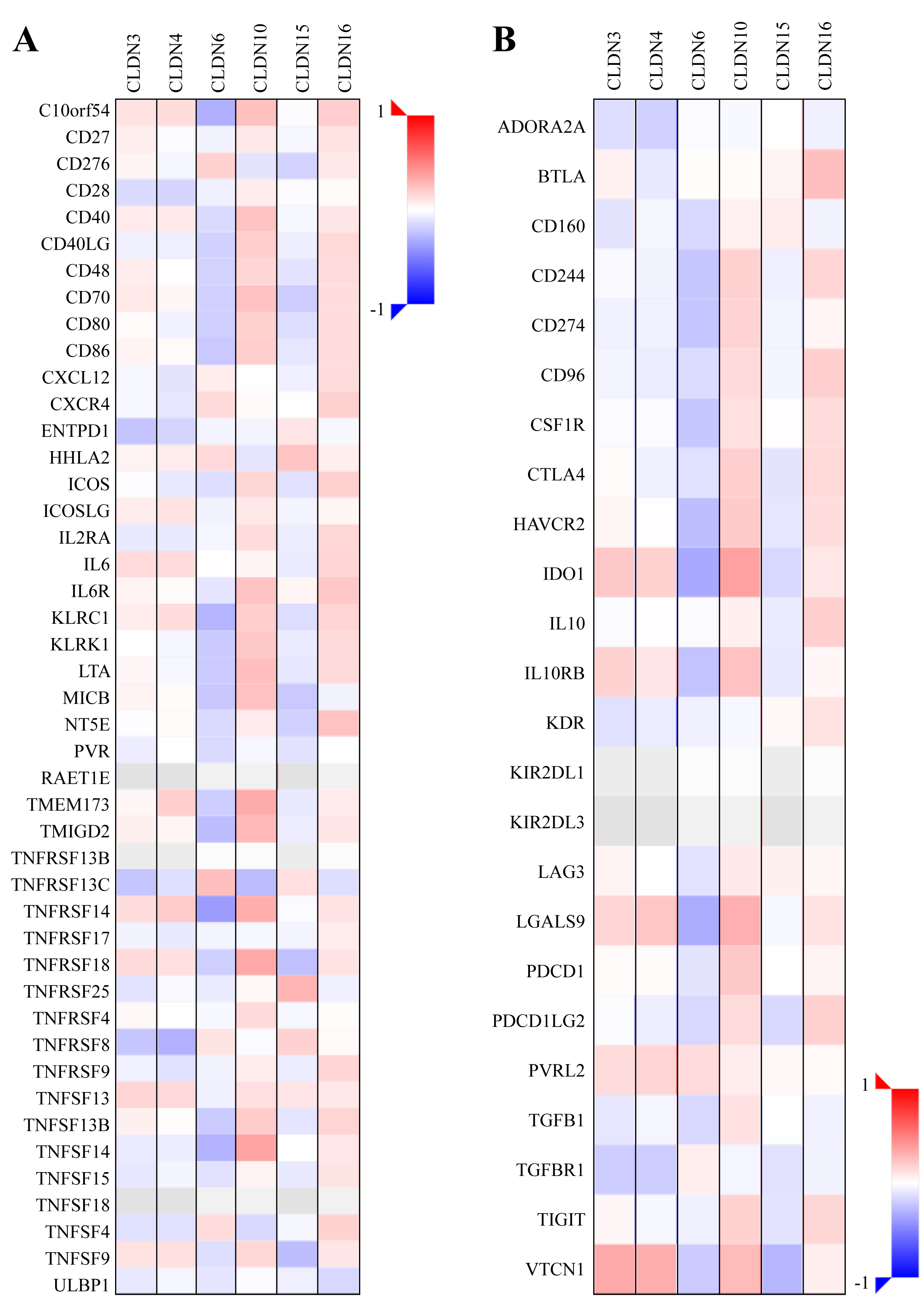

Supplement: Supplementary file 5 [file Image_5.TIF]

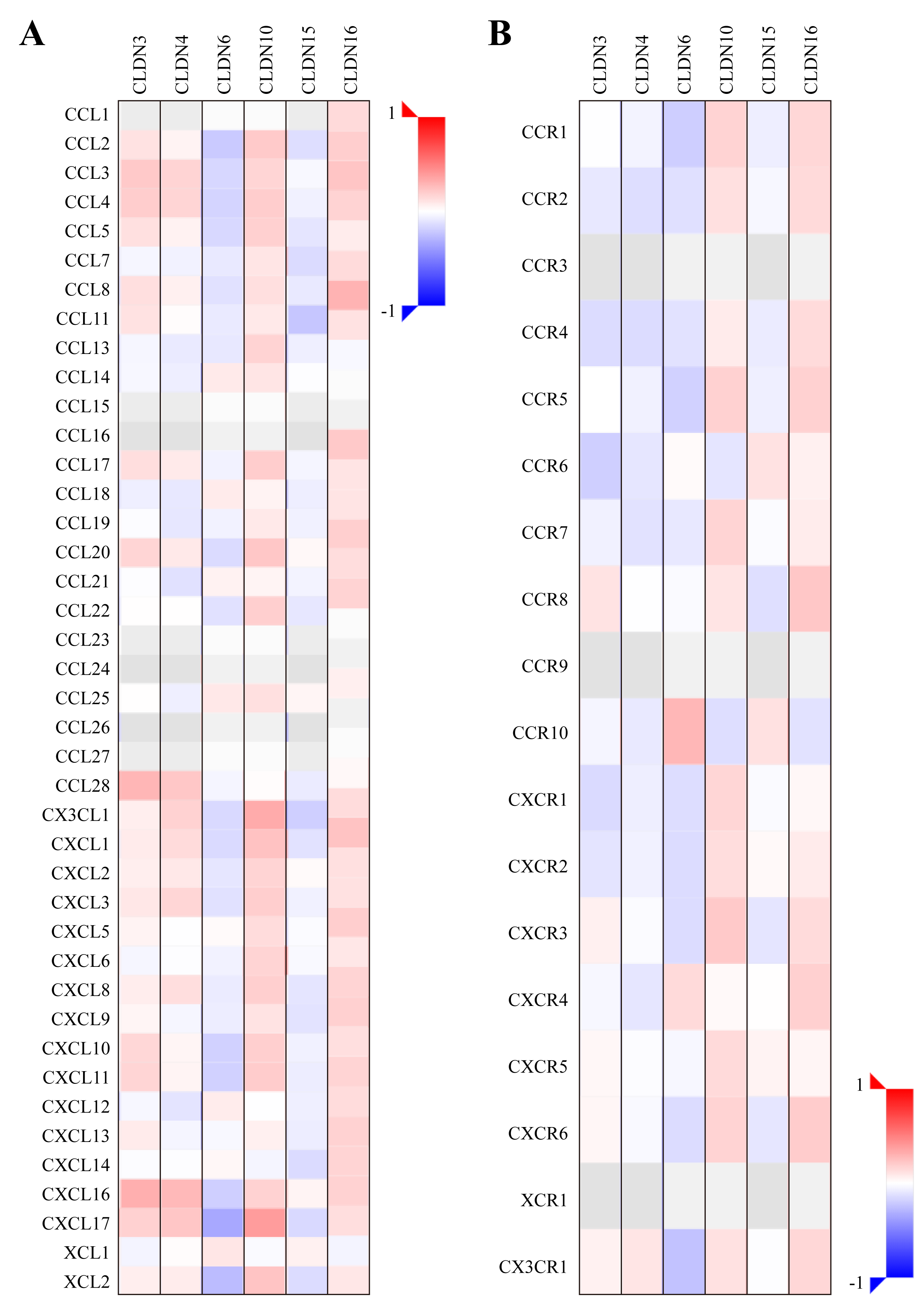

Supplement: Supplementary file 6 [file Image_6.TIF]
